# Supplementary material for: scRNA-seq reveals elevated interferon responses and TNF-α signaling via NFkB in monocytes in children with uncomplicated malaria
Source: Exp Biol Med (Maywood). 2025 Jan 3;249:10233. doi: 10.3389/ebm.2024.10233 (PMC11738629; doi:10.3389/ebm.2024.10233)
Supplement: Supplementary file 1 [file DataSheet1.PDF]

Supplementary Information

Description of Supplementary Tables

Supplementary Table 1: Descriptive statistics of all study participants

Supplementary Table 2: Descriptive statistics of matched study participants for scRNA-Seq

Supplementary Table 3: Dirichlet regression analysis of proportions of various cell types compared between the two groups

Supplementary Table 4: Differentially expressed genes between patients and controls.

Supplementary Table 5: Pathway analysis of differentially expressed genes between patients and control group

Supplementary Table 6: Leading pathways in cell-cell interaction analysis for the patient group

Supplementary Table 7: Leading pathways in cell-cell interaction analysis for the control group

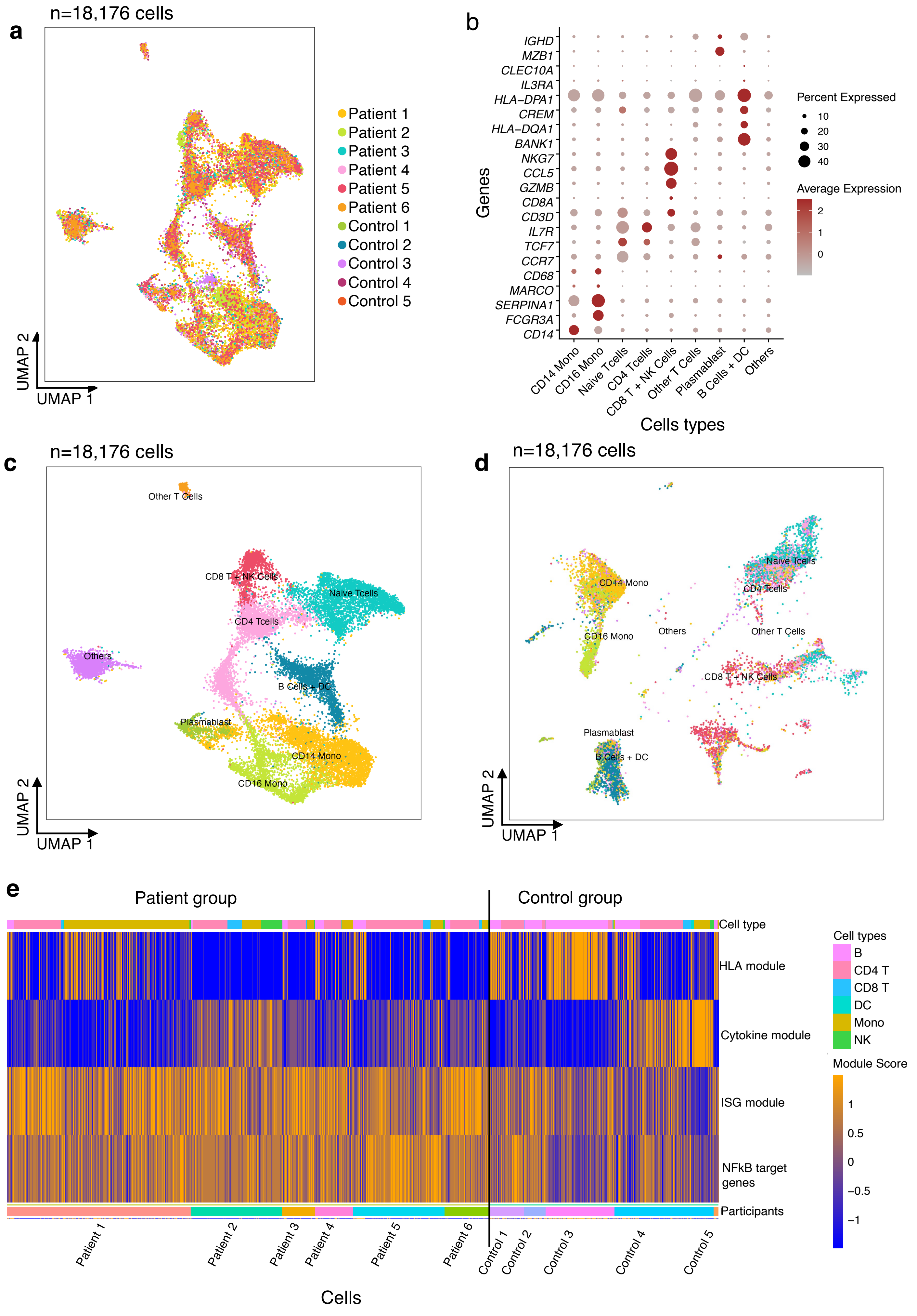
a
n=18,176 cells
Patient 1
Patient 2
Patient 3
Patient 4
Patient 5
Patient 6
Control 1
Control 2
Control 3
Control 4
Control 5
UMAP 2
UMAP 1
b
Genes
IGHD
MZB1
CLEC10A
IL3RA
HLA−DPA1
CREM
HLA−DQA1
BANK1
NKG7
CCL5
GZMB
CD8A
CD3D
IL7R
TCF7
CCR7
CD68
MARCO
SERPINA1
FCGR3A
CD14
CD14 Mono
CD16 Mono
Naive Tcells
CD4 Tcells
CD8 T + NK Cells
Other T Cells
Plasmablast
B Cells + DC
Others
Cells types
Percent Expressed
10
20
30
40
Average Expression
2
1
0
c
n=18,176 cells
Other T Cells
CD8 T + NK Cells
Naive Tcells
CD4 Tcells
Others
B Cells + DC
Plasmablast
CD14 Mono
CD16 Mono
UMAP 2
UMAP 1
d
n=18,176 cells
Naive Tcells
CD14 Mono
CD4 Tcells
CD16 Mono
Others
Other T Cells
CD8 T + NK Cells
Plasmablast
B Cells + DC
UMAP 2
UMAP 1
e
Patient group
Control group
Cell type
HLA module
Cytokine module
ISG module
NFkB target genes
Participants
Cell types
B
CD4 T
CD8 T
DC
Mono
NK
Module Score
1
0.5
0
−0.5
−1
Patient 1
Patient 2
Patient 3
Patient 4
Patient 5
Patient 6
Control 1
Control 2
Control 3
Control 4
Control 5
Cells

Supplementary Figure 1 | Cluster identification and annotation. a) UMAP of 22,819 cells from all participants, showing 10 clusters in the dataset (following iterative Louvain clustering). **b)** Dot plots showing genes used to manually annotate the clusters and show the fraction of cells expressing it and the non-zero expression. Dot size represents the fraction of cell types (rows) expressing a given gene (columns). The dot color indicates scaled average expression by gene column. **c**) UMAP colored by various manually annotated clusters based on the cell markers **d)** UMAP showing cell clusters identified from a reference-mapped dataset but labeled with the manually annotated cluster identities. **e)** Heatmap showing overall module score for each cell and grouped based on each participant and all the cell types, and overall study groups. The color scheme represents a scale for module scores.
